# Factors That Influence the Use of eHealth in Home Care: Scoping Review and Cross-sectional Survey

**DOI:** 10.2196/41768

**Published:** 2023-03-09

**Authors:** Elke Mathijssen, Wendela de Lange, Nienke Bleijenberg, Thijs van Houwelingen, Tiny Jaarsma, Jaap Trappenburg, Heleen Westland

**Affiliations:** 1 The Healthcare Innovation Center Julius Center for Health Sciences and Primary Care University Medical Center Utrecht Utrecht Netherlands; 2 Department of Nursing Science Julius Center for Health Sciences and Primary Care University Medical Center Utrecht Utrecht Netherlands; 3 Research Group Proactive Care for Older People Living at Home University of Applied Sciences Utrecht Utrecht Netherlands; 4 Research Group Technology for Innovations in Healthcare University of Applied Sciences Utrecht Utrecht Netherlands; 5 Department of Health, Medicine and Caring Sciences Linköping University Linköping Sweden

**Keywords:** eHealth, digital health, mHealth, mobile applications, telehealth, telemedicine, telecare, implementation, influencing factors, home care

## Abstract

**Background:**

In home care, eHealth implementation requires health care professionals and home care clients to change their behavior because they have to incorporate the use of eHealth into their daily routines. Knowledge of factors that influence the use of eHealth in home care is needed to optimize implementation strategies. However, a comprehensive overview of such factors is lacking.

**Objective:**

The aims of this study were to (1) provide insight into the types of eHealth that are used and preferred in home care and (2) identify factors that influence the use of eHealth in home care according to health care professionals and home care clients.

**Methods:**

A scoping review and online, cross-sectional survey were conducted sequentially. The survey was conducted among Dutch health care professionals with a nursing background who were working for a home care organization at the time. The capability, opportunity, motivation, behavior (COM-B) model, which posits that for any behavior (B) to occur, a person must have the capability (C), opportunity (O), and motivation (M) to perform the behavior, was used to identify influencing factors. The use of a theoretical model may contribute to a better understanding of how to achieve and sustain behavior change in clinical practice.

**Results:**

We included 30 studies in the scoping review. The most frequently studied type of eHealth was a telecommunication/telemonitoring system. The survey was completed by 102 participants. The most frequently used types of eHealth were electronic health records, social alarms, and online client portals. A health app was the most frequently preferred type of eHealth. We identified 22 factors that influence the use of eHealth in home care according to health care professionals and home care clients. Influencing factors were organized into the components of the COM-B model, namely capability (n=6), opportunity (n=10), and motivation (n=6). We found that there is no single influencing factor that is key to the complexity of eHealth implementation.

**Conclusions:**

Different types of eHealth are used, and many types of eHealth are preferred by health care professionals. The identified factors that influence the use of eHealth in home care relate to all components of the COM-B model. These factors need to be addressed and embedded in implementation strategies of eHealth to optimize the use of eHealth in home care.

## Introduction

Driven by an aging population and rising number of people with one or more chronic conditions, health care systems are moving away from institutional care toward home care [1-4]. Home care, defined as all nursing care provided by nurses and nurse assistants at people’s homes, has proven to be a lower-cost alternative to institutional care in the long term [5]. Furthermore, home care clients experience a higher quality of life than people living in institutional care [6,7]. At the same time, home care faces challenges to keep up with its continued growth, with staffing shortages being the most prominent challenge [8,9].

These challenges are an important driver behind the proliferation of eHealth in home care. eHealth refers to the use of information and communication technologies (ICT) in support of health and health-related fields [10]. Well-known examples of eHealth are electronic health records, online client portals, and health apps. There is a large body of evidence showing the potential of eHealth to significantly contribute to the efficacy, safety, and quality of care [11-14]. However, its use in clinical practice remains limited due to implementation difficulties, including a lack of understanding of what works and does not work in the health care environment in which eHealth is to be implemented [15-17].

In home care, eHealth implementation requires health care professionals and home care clients to change their behavior because they have to incorporate the use of eHealth into their daily routines. Knowledge of factors that influence the use of eHealth in home care is needed to optimize implementation strategies. However, a comprehensive overview of such factors is lacking. Previous studies have provided an incomplete picture by focusing only on a specific type of eHealth (eg, electronic medication dispensers) or subgroup of users (eg, people with dementia) [18,19]. Furthermore, these studies did not use a theoretical model of behavior change. The use of a theoretical model may contribute to a better understanding of how to achieve and sustain behavior change in clinical practice. A wide variety of theoretical models has been developed. Many of these models focus on understanding or predicting intra-individual behavior and occasionally interpersonal factors of behavior rather than understanding behavior change in complex environments in which the behavior occurs [20,21].

In this study, we used the capability, opportunity, motivation, behavior (COM-B) model to identify factors that influence the use of eHealth in home care. The COM-B model posits that, for any behavior (B) to occur, a person must have the capability (C), opportunity (O), and motivation (M) to perform the behavior [22]. Therefore, the components of the COM-B model (capability, opportunity, and motivation) may serve as targets for behavior change interventions [22]. The aims of this study were to (1) provide insight into the types of eHealth that are used and preferred in home care and (2) identify factors that influence the use of eHealth in home care according to health care professionals and home care clients.

## Methods

### Design

A scoping review and online, cross-sectional survey were conducted sequentially to provide insight into the types of eHealth that are used and preferred in home care and identify factors that influence the use of eHealth in home care according to health care professionals and home care clients. The results of the scoping review informed the development of the survey, which provided more detailed data from the perspective of health care professionals in the Netherlands. The PRISMA-ScR (Preferred Reporting Items for Systematic Reviews and Meta-Analyses Extension for Scoping Reviews) checklist and Checklist for Reporting Results of Internet E-Surveys (CHERRIES) were used to guide the reporting of this study [23,24].

### Ethical Considerations

This study did not fall under the scope of the Dutch Medical Research Involving Human Subjects Act (WMO). It therefore did not require approval from an accredited medical ethics committee in the Netherlands. Web-based informed consent was obtained from all participants prior to study participation. All study data were deidentified to protect the privacy and confidentiality of participants. 

### Scoping Review

The 5 stages of the methodological framework for scoping reviews by Arksey and O’Malley [25] and additional recommendations from Levac et al [26] were followed: (1) identifying the research question; (2) identifying relevant studies; (3) study selection; (4) charting the data; and (5) collating, summarizing, and reporting the results.

#### Stage 1: Identifying the Research Question

Corresponding to the aims of this study, the research questions were “What types of eHealth are used and preferred in home care?” and “What factors influence the use of eHealth in home care according to health care professionals and home care clients?”

#### Stage 2: Identifying Relevant Studies

The databases PubMed, Embase, The Cochrane Library, CINAHL, and PsycINFO were initially searched in January 2020, and an update was conducted in April 2021. The PubMed syntax was developed first and then adapted for the other databases (Multimedia Appendix 1). Reference lists of studies found through the database search were hand-searched, and grey literature was searched for unpublished research to ensure completion. The searches were limited to studies published from January 2012. The choice for this date safeguarded the generalizability of our results, as research on eHealth has evolved significantly in recent years. Additionally, studies had to be reported in Dutch or English with full text available.

#### Stage 3: Study Selection

The search results were imported into the online screening tool Rayyan [27]. Duplicates were removed. Studies were screened on title and abstract by 2 researchers (EM and WdL) independently. Each study was assigned a label of “include,” “exclude,” or “undecided.” Studies labeled as “undecided” and discrepancies between the researchers were resolved through a consensus discussion. Subsequently, the full texts of studies labeled as “include” were assessed for eligibility against a set of inclusion and exclusion criteria by 2 researchers (EM and WdL) independently. Studies were included if they were conducted among health care professionals or adult (>18 years old) home care clients. They also had to have outcomes on experiences with or barriers and facilitators to the use of eHealth in home care. We excluded studies set in specialized care (eg, mental health services), hospital-at-home programs, or non-Western countries. Nonempirical, intervention, or n of 1 studies were also excluded.

#### Stage 4: Charting the Data

A data chart was developed by the research team to extract the first author, year of publication, country, design, participants, type of eHealth, and results for each study. Additionally, the mixed methods appraisal tool (MMAT) was used for quality appraisal [28]. Two researchers (EM and WdL) extracted part of the data and checked each other’s work. A consensus meeting was held to resolve discrepancies between the researchers.

#### Stage 5: Collating, Summarizing, and Reporting the Results

We singled out relevant data using the data chart. Influencing factors were organized into the components of the COM-B model, namely capability, opportunity, and motivation. This was done by 3 researchers (EM, WdL, and HW) during joint work sessions.

### Survey

The survey was conducted from June 2020 to August 2020 among Dutch health care professionals with a nursing background who were working for a home care organization at the time. A convenience sampling approach was used to recruit participants through the online newsletter of the Dutch nurses’ association. Additionally, the professional network of the research team was used to recruit participants by email and social media. Participants were encouraged to share the survey with colleagues.

The survey was developed by 3 researchers (EM, WdL, and HW) using the results of the scoping review’s initial search in January 2020. We formulated 20 questions with close and open-ended response formats (Multimedia Appendix 2). Participants had to answer each question to continue through the survey. Qualtrics version 2020 (Qualtrics) was used to place the survey online. The research team pretested the survey to check for potential issues with the questions and response formats or technical glitches. The survey took approximately 15 minutes to complete. Participants who did not complete the survey were excluded from the analysis. Close-ended questions were analyzed with descriptive statistics, using SPSS version 26 (IBM Corporation). Open-ended questions were analyzed descriptively to enrich the quantitative data. The survey was anonymous. We did not collect personal data such as names and addresses. Data were handled according to the Dutch implementation act of the General Data Protection Regulation.

## Results

### Scoping Review

**Figure 1:**
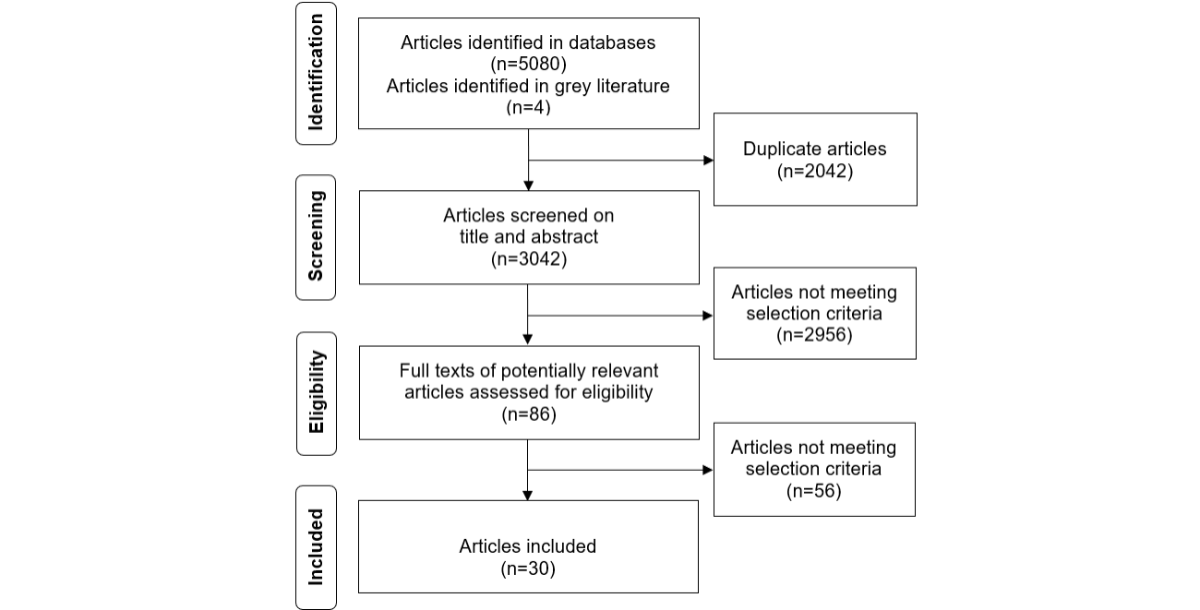
Flow diagram of the study selection process.

The results of the initial search in January 2020 and update in April 2021 were merged. The results of the update affirmed those of the initial search and did not provide any new insights. In total, 30 studies were included [19,29-57]. Figure 1 shows a flow diagram of the study selection process.

### Study Characteristics

The majority of the studies were conducted in the United States (8/30, 27%), Norway (7/30, 23%), or the Netherlands (6/30, 20%). The studies had a quantitative (5/30, 17%), qualitative (19/30, 63%), or mixed methods (6/30, 20%) design. Participants were health care professionals (17/30, 57%), home care clients (6/30, 20%), or both (7/30, 23%). Table 1 shows an overview of the studies’ characteristics. All criteria in the MMAT were met by 1 study with a quantitative design, 14 studies with a qualitative design, and 1 study with a mixed methods design (Multimedia Appendix 3).

**Table 1:** An overview of the studies’ characteristics.

First author (year of publication)	Country	Design	Aim	Participants	Type of eHealth
Peeters (2012) [42]	The Netherlands	Quantitative	(1) To gain insight into individual client characteristics and characteristics of home telecare, which influence the adoption of home telecare by older or chronically ill clients of home care organizations in the Netherlands or (2) To examine the applicability of Rogers’ concept of “perceived attributes” in explaining the factors that might influence the decision to adopt home telecare	Home care clients	Telecommunication/telemonitoring
Postema (2012) [43]	The Netherlands	Qualitative	To determine which factors influence the success of the implementation of video communication as a home telecare application from an organizational perspective	Health care professionals and home care clients	Telecommunication/telemonitoring system
Radhakrishnan (2012) [47]	United States	Qualitative	(1) To explore perceptions on effectiveness of telehealth for heart failure (HF) management and (2) to explore facilitators and barriers to continued use of telehealth by patients with HF and their nurses beyond the initial acceptance phase in a home care setting	Health care professionals and home care clients	Telecommunication/telemonitoring system
Brody (2013) [49]	United States	Mixed methods	To examine the feasibility of a web-based education program to help nurses improve their treatment of geriatric pain and depression	Health care professionals	Online education program
Nielsen (2013) [41]	Denmark	Mixed methods	To examine a large-scale government-sponsored mobile health implementation program in the Danish home care sector and to understand how the technology was used differently across home care agencies	Health care professionals	Health app
Alaiad (2014) [39]	United States	Quantitative	To understand the determinants of home health care robot adoption from social, technical and managerial aspects by applying technology acceptance theories	Health care professionals and home care clients	Medical robot
Sockolow (2014) [40]	United States	Mixed methods	To identify challenges and facilitators to electronic health record (EHR) adoption to inform EHR development and implementation	Health care professionals	EHR
Cai (2015) [36]	Denmark	Qualitative	To explore how health professionals experience and use the intelligent bed in patients’ homes	Health care professionals	Intelligent bed
van Houwelingen (2015) [38]	The Netherlands	Quantitative	To examine predictors of Dutch nurses’ willingness to use home telecare	Health care professionals	Telecommunication/telemonitoring system
Peek (2016) [48]	The Netherlands	Qualitative	To provide insight into the positions of stakeholder groups involved in the implementation of technology for aging in place	Health care professionals and home care clients	Not specified or multiple types of eHealth
Radhakrishnan (2016) [37]	United States	Mixed methods	(1) To explore the reasons for the initial adoption and the eventual decline of a decade-long home telehealth program at a Texas home health agency and (2) to explore barriers to and facilitators for sustaining home telehealth programs	Health care professionals	Telecommunication/telemonitoring system
Stokke (2017) [44]	Norway	Qualitative	To explore how actors who are involved with the social alarm, which is an established technology innovation, relate to, perceive, and articulate the expectations of the technology in everyday living	Home care clients	Social alarm
Göransson (2018) [35]	Sweden	Qualitative	To explore the experiences of using an app for reporting health concerns among older people with home-based health care and their home care nurses	Health care professionals and home care clients	Health app
Nakrem (2018) [52]	Norway	Qualitative	To explore how home health care professionals experienced the introduction of digital medicine dispensers and their influence on patient-caregiver relationships	Health care professionals	Electronic medication dispenser
Øyen (2018) [45]	Norway	Quantitative	To better understand nurses’ and other staff members’ attitudes toward the usefulness of information and communication technology in home care	Health care professionals	Not specified or multiple types of eHealth
van Doorn-van Atten (2019) [50]	The Netherlands	Mixed methods	To evaluate the feasibility of a telemonitoring intervention to improve the nutritional status of community-dwelling older adults	Health care professionals and home care clients	Telecommunication/telemonitoring system
Funderskov (2019) [33]	Denmark	Qualitative	To explore the advantages and disadvantages of using video consultations, as experienced by specialized palliative care health care professionals who are involved in palliative care at home	Health care professionals	Telecommunication/telemonitoring system
Ibrahim (2019) [46]	Canada	Qualitative	To explore nurses' experiences with electronic documentation system usage in the home care sector	Health care professionals	EHR
Johannessen (2019) [31]	Norway	Qualitative	To explore home care professionals' perceptions of safety related to the use of telecare by older adults	Health care professionals	Telecommunication/telemonitoring system
Karlsen (2019) [51]	Norway	Qualitative	To obtain a deeper understanding of the persistent use of telecare for older adults and their family caregivers	Home care clients	Telecommunication/telemonitoring system
Kozikowski (2019) [29]	United States	Qualitative	To gain insight into the perspectives of home-based primary care (HBPC) staff regarding adopting telehealth technology to increase the reach of HBPC to more homebound patients.	Health care professionals	Telecommunication/telemonitoring system
Rosborg (2019) [30]	Sweden	Qualitative	To study different mobile health (mHealth) tools used in both countries and try and possibly improve mHealth tools and how they contribute to health care delivery	Health care professionals	Health app
Rydenfält (2019) [34]	Sweden	Qualitative	To gain a broader understanding of how eHealth currently can be used in home care nursing and how home care nursing providers imagine its future potential	Health care professionals	Not specified or multiple types of eHealth
Seto (2019) [32]	Canada	Qualitative	To determine the feasibility of implementing a mobile phone–based telemonitoring system through a home care nursing agency and to explore the feasibility of conducting a future effectiveness trial	Health care professionals and home care clients	Health app
Glomsås (2020) [56]	Norway	Qualitative	To learn more about factors that promote or inhibit user involvement among health professionals when implementing welfare technology in home care services	Health care professionals	Not specified or multiple types of eHealth
Kivekäs (2020) [55]	Finland	Quantitative	To describe the factors that determine a user’s intent to adopt new welfare technologies in the context of home care	Health care professionals	Not specified or multiple types of eHealth
Woo (2020) [57]	United States	Qualitative	To investigate factors that affect the decision to adopt or decline telehealth at home among patients with HF	Home care clients	Telecommunication/telemonitoring system
Birkhoff (2021) [53]	United States	Mixed methods	(1) To explore the facilitators of and challenges with HF patients’ ability to use and potentially adopt a virtual nurse visit (VNV) and (2) to assess their satisfaction and experiences using the VNV in conjunction with traditional in-person home care nursing visits	Home care clients	Telecommunication/telemonitoring system
van der Cingel (2021) [54]	The Netherlands	Qualitative	To get insight into the way home care nurses assess eHealth interventions during assessment of care	Health care professionals	Not specified or multiple types of eHealth
Glomsås (2021) [19]	Norway	Qualitative	To explore elderly service users’ experience with user involvement in the implementation and everyday use of welfare technology in public home care services	Home care clients	Not specified or multiple types of eHealth

### Survey

The survey was completed by 102 participants. Their characteristics are shown in Table 2.

**Table 2:** Characteristics of the participants (n=102).

Characteristics		Results
Age (years), mean (SD)		43 (12)
**Gender, n (%)**		
	Male	8 (7.8)
	Female	93 (91.2)
	Not specified	1 (1)
**Profession, n (%)**		
	Nurse assistant (NLQF^a^ 3)	11 (10.8)
	Vocational level nurse (NLQF 4)	16 (15.7)
	Bachelor level nurse (NLQF 6)	58 (56.9)
	Nurse practitioner (NLQF 7)	1 (1)
	Other (eg, nursing student, quality officer, team manager)	15 (14.7)
Work experience (years), mean (SD)		9.7 (9)

^a^NLQF: Dutch qualifications framework.

### What Types of eHealth Are Used and Preferred in Home Care?

The scoping review included studies on 8 different types of eHealth (Table 1). The most frequently studied type of eHealth was a telecommunication/telemonitoring system (12/30, 40%) [29,31,33,37,38,42,43,47,50,51,53,57]. Other studied types of eHealth were a health app (n=4) [30,32,35,41], electronic health record (n=2) [40,46], electronic medication dispenser (n=1) [52], social alarm (n=1) [44], medical robot (n=1) [39], intelligent bed (n=1) [36], and online education program (n=1) [49]; 7 studies did not specify the type of eHealth under study or studied multiple types of eHealth [19,34,45,48,54-56].

In the survey, the majority of the participants (94/102, 92.1%) indicated that eHealth is currently used within their organization. The most frequently used types of eHealth were electronic health records (92/102, 90.2%), social alarms (83/102, 81.4%), and online client portals (76/102, 74.5%). Most of the participants (78/102, 76.5%) preferred more use of eHealth within their organization. A health app was the most frequently preferred type of eHealth (55/102, 53.9%). Table 3 shows the types of eHealth that were used and preferred by the participants.

**Table 3:** The types of eHealth that were used and preferred by the participants (n=102).

Type of eHealth^a^	Used, n (%)	Preferred, n (%)
Electronic health record	92 (90.2)	3 (2.9)
Social alarm	83 (81.4)	1 (1)
Online client portal	76 (74.5)	8 (7.8)
Email and/or chat consultations	55 (53.9)	13 (12.7)
Electronic medication dispenser	52 (51)	26 (25.5)
Video consultations	46 (45.1)	39 (38.2)
Electronic door lock	17 (16.7)	30 (29.4)
(Portable) sensor	17 (16.7)	33 (32.3)
Health app	12 (11.8)	55 (53.9)
Medical robot	11 (10.8)	34 (33.3)
Not applicable	3 (2.9)	9 (8.8)
Other^b^	0 (0)	4 (3.9)

^a^Multiple answers were allowed.

^b^Not specified.

### What Factors Influence the Use of eHealth in Home Care?

**Figure 2:**
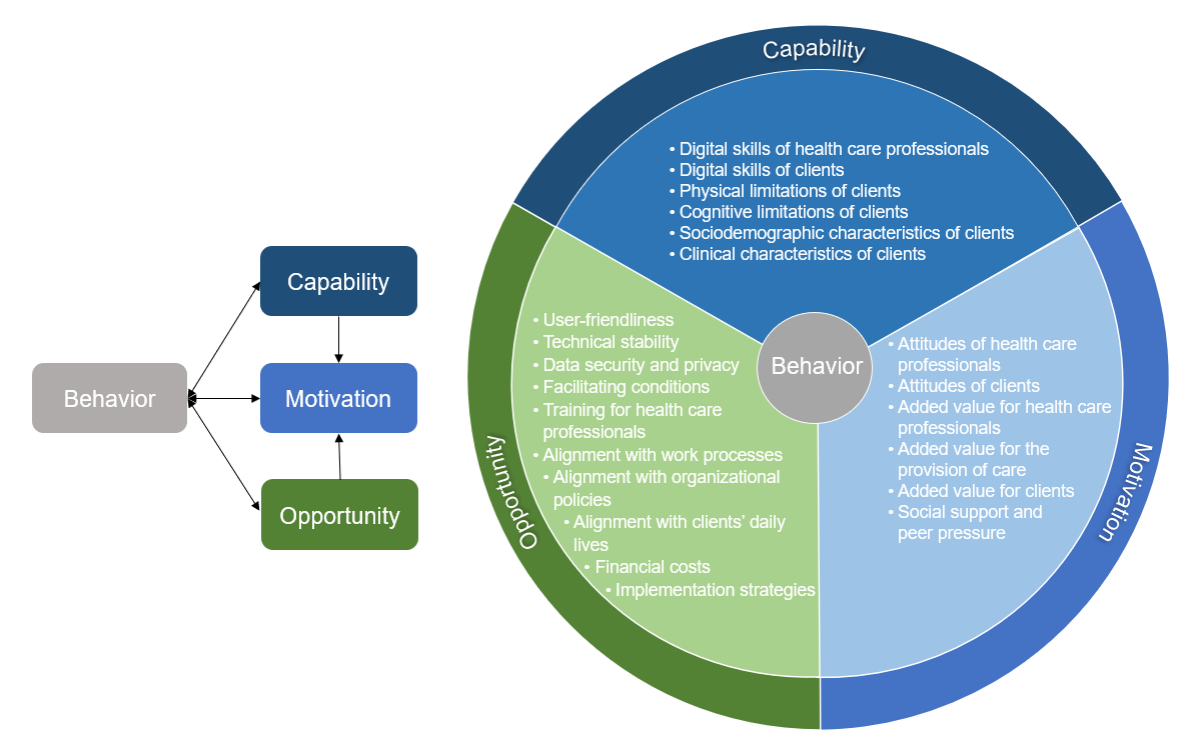
Influencing factors categorized into the COM-B model.

We identified 22 influencing factors and organized these into the components of the COM-B model (capability, opportunity, and motivation; Figure 2).

#### Capability

We identified 6 factors that influence health care professionals’ and home care clients’ capability to use eHealth in home care: (1) digital skills of health care professionals, (2) digital skills of home care clients, (3) physical limitations of home care clients, (4) cognitive limitations of home care clients, (5) sociodemographic characteristics of home care clients, and (6) clinical characteristics of home care clients.

The scoping review showed that health care professionals and home care clients who have sufficient digital skills are more capable of using eHealth than the ones who have not [37,45-47,50,52-54,56,57]. Furthermore, physical limitations of home care clients may negatively influence their capability to use eHealth [32,47]. The same applies to cognitive limitations of home care clients [19,37,44,47,50]. Regarding sociodemographic characteristics of home care clients, the studies mainly focused on age [37,42,47,50]. Older age is associated with a decreased capability to use eHealth. A longer disease duration and higher disease activity were described in the studies as clinical characteristics of home care clients that may negatively influence their capability to use eHealth [37,47,54].

The survey showed that most of the participants (60/102, 58.8%) considered their knowledge and skills to use eHealth sufficient. The vast majority of the participants (84/102, 82.4%) considered themselves fast learners when it comes to the use of eHealth, and 18.6% (19/102) of the participants indicated that they were provided with sufficient training on eHealth during their professional education. Most of the participants (59/102, 57.8%) indicated that they had a current need for training on eHealth. According to 90.2% (92/102) of the participants, some home care clients are not capable of using eHealth. People with dementia were often cited as an example. However, the participants stressed that the capability to use eHealth is determined individually.

#### Opportunity

We identified 10 factors that influence the opportunity of health care professionals and home care clients to use eHealth in home care: (1) user-friendliness, (2) technical stability, (3) data security and privacy, (4) facilitating conditions, (5) training for health care professionals, (6) alignment with work processes of health care professionals, (7) alignment with organizational policies, (8) alignment with home care clients’ daily lives, (9) financial costs, and (10) implementation strategies.

The scoping review showed that user-friendliness and technical stability of a type of eHealth are preconditions for use [19,30-35,37,38,40-43,46-52,54,56,57]. Furthermore, data security and privacy must be considered from the very outset [36,37,39,48]. Facilitating conditions (eg, the provision of resources such as computers, tablets, and smartphones) and training for health care professionals may positively influence health care professionals’ opportunity to use eHealth [36,37,39,40,43-48,50,54-57]. Health care professionals’ and home care clients’ opportunity to use eHealth increases if the use of eHealth aligns with work processes of health care professionals and organizational policies [29,30,32-34,37,40-44,47-49,52,56]. The same applies to alignment with home care clients’ daily lives [19,32,37,42,43,52]. Financial costs may negatively influence their opportunity to use eHealth [34,40,41,43,48,54]. Regarding implementation strategies, the studies mainly focused on the importance of involving health care professionals and home care clients in the implementation phase of new types of eHealth [34,36,37,40,41,43,47-50,56].

In the survey, 34.3% (35/102) of the participants indicated that the amount of types of eHealth that were currently available was sufficient. Most of the participants (68/102, 66.7%) indicated that they encountered problems with the use of eHealth. Technical issues and financial costs were at the top of the list (51/102, 50% and 36/102, 35.3%, respectively). Of the participants, 34.3% (35/102) indicated that their organization encouraged employees “always” or “often” to use eHealth, and 48% (49/102) of the participants indicated that their organization was “always” or “often” open to initiatives by employees regarding the use of eHealth. Furthermore, 40.2% (41/102) of the participants indicated that their organization “always” or “often” involved employees in the implementation phase of new types of eHealth, and 23.5% (24/102) of the participants indicated that their organization “always” or “often” involved home care clients in the implementation phase of new types of eHealth.

#### Motivation

We identified 6 factors that influence health care professionals’ and home care clients’ motivation to use eHealth in home care: (1) attitudes of health care professionals, (2) attitudes of home care clients, (3) added value for health care professionals, (4) added value for the provision of care, (5) added value for home care clients, and (6) social support and peer pressure.

The scoping review showed that attitudes of health care professionals and home care clients are shaped by their beliefs and prior experiences regarding eHealth [35-41,43,44,46-48,52-54,57]. Health care professionals and home care clients who have positive beliefs and prior experiences regarding eHealth are more motivated to use eHealth than those who have not. Health care professionals’ motivation to use eHealth increases if the use of eHealth is of added value for them (eg, increased work efficiency) [29,32-37,40-42,46,47,49,52]. It is also motivating for health care professionals if the use of eHealth is of added value for the provision of health care (eg, decreased health care utilization) and home care clients (eg, increased safety) [19,29,31-33,35-39,41-45,47,48,50-55]. Furthermore, health care professionals’ and home care clients’ motivation to use eHealth may increase or decrease due to social support and peer pressure [37,39,44,47,51,57]. For example, health care professionals are more motivated to use eHealth when they see their colleagues doing so.

The survey showed that most of the participants (69/102, 67.6%) thought that the use of eHealth had more benefits than drawbacks. The majority of the participants (90/102, 88.2%) thought that the use of eHealth saved time and costs. Of the participants, 70.6% (72/102) thought that the use of eHealth increased the overall quality of care. Furthermore, they thought that the use of eHealth increased home care clients’ self-reliance and safety (93/102, 91.2% and 87/102, 85.3%, respectively). Of the participants, 61.8% (63/102) thought that the use of eHealth did not compromise personal contact between health care professionals and home care clients. Most (74/102, 72.5%) of the participants (completely) agreed with the statement “I have confidence in the advent of new types of eHealth.” Correspondingly, 69.6% (71/102) of the participants indicated that they were not afraid of losing their job or job activities due to the advent of new types of eHealth. In addition, 7.8% (8/102) of the participants (completely) agreed with the statement “I am not looking forward to the advent of new types of eHealth,” and 26.5% (27/102) of the participants (completely) agreed with the statement “Home care clients are not looking forward to the advent of new types of eHealth.” The participants stressed that the use of eHealth should never be made an obligation for home care clients.

## Discussion

### Principal Findings

The aims of this study were to provide insight into the used and preferred types of eHealth in home care and identify factors that influence the use of eHealth in home care according to health care professionals and home care clients. Our results show that different types of eHealth such as electronic health records, social alarms, and online client portals are used in home care. However, there are also many preferred types of eHealth in home care (eg, health apps). This indicates that there is substantial room for improvement when it comes to eHealth implementation. We identified 22 factors that influence the use of eHealth in home care according to health care professionals and home care clients. We found that there is no single influencing factor that is key to the complexity of eHealth implementation. Influencing factors relate to all components of the COM-B model (ie, capability, opportunity, and motivation), which interact to generate behavior. Therefore, factors that influence the use of eHealth in home care can be considered diffuse and intertwined.

### Comparisons With Prior Work

Our results compare with those of studies on factors that influence the use of eHealth in other settings than home care. Influencing factors that were identified in a review of reviews by Lau et al [58] in primary care are among others providing evidence of benefit, facilitating conditions, and costs. Comparable results were found in a review of reviews by Ross et al [59] and a systematic review with expert discussions by Schreiweis et al [60]. Both studies were not limited to a particular setting. The comparability of results between studies indicates that influencing factors are generalizable across settings. In this study, no influencing factors unique to home care were identified. Furthermore, our results show that factors that influence the use of eHealth in home care remain considerably constant over time. Indeed, we found little to no variation between the results of the studies included in the scoping review despite publication years ranging from 2012 to 2021.

In the survey, the participants stressed that the use of eHealth should always be a free choice instead of an obligation for home care clients. This fits with the fundamentals of person-centered care in which an individual is put centrally and his or her needs, preferences, and values are the driving force of all health care decisions [61]. Previous studies have shown that eHealth has the potential to support person-centered care [62-64]. For example, electronic health records, online client portals, and health apps may provide home care clients with reliable and timely health information and empower them to take an active role in their own care. Other types of eHealth such as telecommunication/telemonitoring systems may facilitate a trusting professional care relationship. However, the belief that the use of eHealth compromises personal contact between health care professional and home care clients is common. Health care professionals generally strive to work in a person-centered way [54]. Therefore, it is important to provide them with evidence regarding eHealth’s potential to support person-centered care. This may contribute to higher adoption rates.

A qualitative study by Korpershoek et al [65] in the field of chronic obstructive pulmonary disease showed that eHealth is more readily accepted when tailored to individual needs, which stresses the importance of personalization over a one-size-fits-all approach. Therefore, the involvement of health care professionals and home care clients in the implementation phase of new types of eHealth is indispensable. Our results show that this rarely happens today. To enhance the use of eHealth in home care, we suggest, for starters, that home care organizations listen to the needs of the intended users and then translate what is heard into implementation strategies. The use of theory-based implementation instruments (eg, the eHealth Implementation Toolkit) may facilitate the involvement of health care professionals and home care clients in the implementation phase of new types of eHealth [66]. Additionally, influencing factors should be targeted when developing behavior change interventions and organizational policies. Linking the COM-B model to the Behavior Change Wheel by Michie et al [22] allows for a systematic approach to transit influencing factors to behavior change interventions and organizational policies that are likely to be effective in achieving and sustaining behavior change in clinical practice.

### Strengths and Limitations

This study was carefully designed and conducted. We applied several methods to enhance its quality such as the use of reporting checklists; a comprehensive search strategy; and dual, independent screening for the study selection process. An extensive search of the literature indicated that this study is the first to use the COM-B model to identify factors that influence the use of eHealth in home care. We considered the use of an established theoretical underpinning from the behavior change literature as an important strength. Although the COM-B model was initially applied to intervention design, it is now increasingly applied as a solid synthesis framework by studies in various contexts [67,68]. Our study confirms that the COM-B model can be applied as such. There are also some limitations that need to be considered. Despite our attempt to be as inclusive as possible, the scoping review may have missed some relevant studies due to language restrictions. Furthermore, many types of eHealth are commercially developed and marketed. The literature was limited to those that have undergone scientific evaluation.

The survey was completed by health care professionals with a diverse nursing educational background, including nurse assistants, nurses with a vocational or bachelor’s degree, and nurse practitioners within the specific context of the Dutch home care system. These results reflect the Dutch context; however, the generalizability of these results might be limited to home care in countries with a similar home care system. Moreover, the focus of this study was largely on the perspective of health care professionals. Future studies with a larger focus on the perspective of home care clients are warranted to expose this key stakeholder’s voice.

### Conclusions

In home care, different types of eHealth are used, and many types of eHealth are preferred by health care professionals*.* We identified 22 factors that influence the use of eHealth in home care and organized these factors into the components of the COM-B model. Influencing factors relate to all components of the COM-B model, including capability, opportunity, and motivation. Factors intertwine, and no factor is key to cover the complexity of eHealth implementation. To optimize the use of eHealth in home care, these factors need to be addressed and embedded in implementation strategies of eHealth in home care.

## Appendix

Multimedia Appendix 1 PubMed syntax.

Multimedia Appendix 2 Original Dutch survey.

Multimedia Appendix 3 Quality appraisal.

## Abbreviations

CHERRIES: Checklist for Reporting Results of Internet E-Surveys
COM-B: capability, opportunity, motivation, behavior
ICT: information and communication technologies
MMAT: mixed methods appraisal tool
PRISMA-ScR: Preferred Reporting Items for Systematic Reviews and Meta-Analyses Extension for Scoping Reviews
WMO: Medical Research Involving Human Subjects Act
