# Supplementary material for: Factors That Influence the Use of eHealth in Home Care: Scoping Review and Cross-sectional Survey
Source: J Med Internet Res. 2023 Mar 9;25:e41768. doi: 10.2196/41768 (PMC10037173; doi:10.2196/41768)
Supplement: Multimedia Appendix 1 [file jmir_v25i1e41768_app1.docx]

**Multimedia Appendix 1. PubMed syntax.**

| **PubMed (Limit: FROM 1 JANUARY 2012) = 1.010 hits** | |
| --- | --- |
| **#1** | Home Care Agencies [MeSH] OR Home Care Services [MeSH] OR Home Care Services, Hospital-Based [MeSH] OR Home Health Nursing [MeSH] OR Home Health Aides [MeSH] OR “Home Care” OR Homecare OR “Home Health” OR “Home Healthcare” OR Home-based |
| **#2** | Telemedicine [MeSH] OR Telemedicine OR Tele-medicine OR Telecare OR Tele-care OR Telehealth OR Tele-health OR Telehealthcare OR Tele-healthcare OR Telenursing OR Tele-nursing OR Teleintervention OR Tele-intervention OR “Digital Health” OR “Digital Healthcare” OR “Electronic Health” OR “Electronic Healthcare” OR Ehealth OR E-health OR “Mobile Health” OR Mhealth OR M-health OR “Wireless health” OR “Information technology” OR “Communication technology” OR ICT |
| **#3** | Barrier* [tiab] OR Facilitat* [tiab] OR Hinder* [tiab] OR Enabl* [tiab] OR Fail* [tiab] OR Succes* [tiab] OR Experienc* [tiab] OR Challeng* [tiab] OR Implication*[tiab] OR “Lessons Learned” [tiab] OR Implement* [tiab] OR Integrat* [tiab] OR Adopt* [tiab] |
| **#4** | #1 AND #2 AND #3 |
