# Supplementary material for: Factors That Influence the Use of eHealth in Home Care: Scoping Review and Cross-sectional Survey
Source: J Med Internet Res. 2023 Mar 9;25:e41768. doi: 10.2196/41768 (PMC10037173; doi:10.2196/41768)
Supplement: Multimedia Appendix 2 [file jmir_v25i1e41768_app2.docx]

**Multimedia Appendix 2. Original Dutch survey*.***

***Pagina 1: introductie***

**Welkom!**

Deze vragenlijst gaat over het gebruik van eHealth in de wijkverpleging en bestaat uit 22 vragen. Invullen duurt maximaal 15 minuten. Let op: u kunt de vragenlijst niet afsluiten en op een later moment weer openen. Vul de vragenlijst daarom in één keer in.

Klik op ‘Volgende’ om de vragenlijst te starten.

***Pagina 2: karakteristieken respondent***

1. **Wat is uw leeftijd (in jaren)?**

…

1. **Wat is uw geslacht?**

- Man
- Vrouw
- Anders, namelijk: …

1. **Wat is op dit moment uw functie in de wijkverpleging?**

- Verzorgende
- Verzorgende IG
- Verpleegkundige in de wijk
- Wijkverpleegkundige
- Gespecialiseerd verpleegkundige
- Verpleegkundig specialist
- Teamleider/leidinggevende en meewerkend in de zorg
- Teamleider/leidinggevende en niet-meewerkend in de zorg
- Anders, namelijk: …

1. **Hoe lang werkt u in de wijkverpleging (in jaren)?**

…

1. **In welke provincie levert u/uw team wijkverpleging (meerdere antwoorden mogelijk)?**

- Drenthe
- Flevoland
- Friesland
- Gelderland
- Groningen
- Limburg
- Noord-Brabant
- Noord-Holland
- Overijssel
- Utrecht
- Zeeland
- Zuid-Holland

***Pagina 3: huidige en gewenste situatie (deel 1)***

1. **Wordt er op dit moment gebruik gemaakt van eHealth binnen uw organisatie?**

- Ja
- Nee
- Weet ik niet

1. **Kruis aan welke eHealth toepassingen op dit moment worden gebruikt binnen uw organisatie (meerdere antwoorden mogelijk).**

- Beeldbellen met cliënten
- E-mailen en/of chatten met cliënten
- Elektronisch cliëntendossier
- Cliëntenportaal (d.w.z. een online omgeving waarin cliënten zorg-gerelateerde informatie kunnen inzien)
- Elektronisch deurslot
- (Draagbare) personenalarmering
- (Draagbare) sensor (bijv. voor valdetectie of het meten van vitale functies)
- Elektronische medicijndispenser
- Zorgrobot
- Gezondheidsapp (bijv. voor geheugenondersteuning of zelfmanagement)
- Anders, namelijk: …
- Niet van toepassing

***Pagina 4: huidige en gewenste situatie (deel 2)***

1. **Zou u willen dat er meer gebruik wordt gemaakt van eHealth binnen uw organisatie?**

- Ja
- Waarom wel?

………………………………………………………………………………………………………………………………………………………………………………………………………………………………………………………………………………………………………………………Nee

- Waarom niet?

………………………………………………………………………………………………………………………………………………………………………………………………………………………………………………………………………………………………………………………

1. **Kruis aan welke eHealth toepassingen op dit moment (nog) niet worden gebruikt binnen uw** **organisatie, maar u wel zou willen gebruiken (meerdere antwoorden mogelijk).**

- Beeldbellen met cliënten
- E-mailen en/of chatten met cliënten
- Elektronisch cliëntendossier
- Cliëntenportaal (d.w.z. een online omgeving waarin cliënten zorg-gerelateerde informatie kunnen inzien)
- Elektronisch deurslot
- (Draagbare) personenalarmering
- (Draagbare) sensor (bijv. voor valdetectie of het meten van vitale functies)
- Elektronische medicijndispenser
- Zorgrobot
- Gezondheidsapp (bijv. voor geheugenondersteuning of zelfmanagement)
- Anders, namelijk: …
- Niet van toepassing

***Pagina 5: capaciteit (deel 1)***

1. **Geef aan in hoeverre u het eens bent met de onderstaande uitspraken.**

Ik beschik over voldoende kennis en vaardigheden om eHealth te gebruiken.

Helemaal mee eens – Mee eens – Neutraal – Mee oneens – Helemaal mee oneens

Ik leer snel als het gaat om het gebruik van eHealth.
Helemaal mee eens – Mee eens – Neutraal – Mee oneens – Helemaal mee oneens

Tijdens mijn opleiding ben ik voldoende geschoold in het gebruik van eHealth.
Helemaal mee eens – Mee eens – Neutraal – Mee oneens – Helemaal mee oneens

Ik heb behoefte aan scholing in het gebruik van eHealth.

Helemaal mee eens – Mee eens – Neutraal – Mee oneens – Helemaal mee oneens

Ik laat het gebruik van eHealth liever aan mijn collega’s over.
Helemaal mee eens – Mee eens – Neutraal – Mee oneens – Helemaal mee oneens

***Pagina 6: capaciteit (deel 2)***

1. **Zijn cliënten die wijkverpleging ontvangen volgens u voldoende in staat om eHealth te gebruiken?**

- Ja
- Sommige cliënten wel, sommige cliënten niet
- Welke cliënten wel en welke cliënten niet? Kunt u uitleggen hoe dat komt?

………………………………………………………………………………………………………

………………………………………………………………………………………………………

………………………………………………………………………………………………………

- Nee
- Kunt u uitleggen hoe dat komt?

………………………………………………………………………………………………………

………………………………………………………………………………………………………

………………………………………………………………………………………………………

***Pagina 7: motivatie (deel 1)***

1. **Geef aan in hoeverre u het eens bent met de onderstaande uitspraken.**

Ik heb vertrouwen in (nieuwe) eHealth toepassingen.

Helemaal mee eens – Mee eens – Neutraal – Mee oneens – Helemaal mee oneens

Er is voldoende aanbod aan (nieuwe) eHealth toepassingen.

Helemaal mee eens – Mee eens – Neutraal – Mee oneens – Helemaal mee oneens

Ik zie meer voordelen dan nadelen aan het gebruik van eHealth.

Helemaal mee eens – Mee eens – Neutraal – Mee oneens – Helemaal mee oneens

Ik zit niet te wachten op de komst van (nieuwe) eHealth toepassingen.

Helemaal mee eens – Mee eens – Neutraal – Mee oneens – Helemaal mee oneens

Het gebruik van eHealth kan tijd en/of kosten besparen.

Helemaal mee eens – Mee eens – Neutraal – Mee oneens – Helemaal mee oneens

Ik ben bang om mijn werk of een deel van (de inhoud van) mijn werkzaamheden kwijt te raken door de komst van (nieuwe) eHealth toepassingen.

Helemaal mee eens – Mee eens – Neutraal – Mee oneens – Helemaal mee oneens

Het gebruik van eHealth kan de werkdruk verhogen.

Helemaal mee eens – Mee eens – Neutraal – Mee oneens – Helemaal mee oneens

***Pagina 8: motivatie (deel 2)***

1. **Geef aan in hoeverre u het eens bent met de onderstaande uitspraken.**

Mijn collega’s staan positief tegenover het gebruik van eHealth.

Helemaal mee eens – Mee eens – Neutraal – Mee oneens – Helemaal mee oneens

Het gebruik van eHealth kan de kwaliteit van zorg verhogen.

Helemaal mee eens – Mee eens – Neutraal – Mee oneens – Helemaal mee oneens

Cliënten zitten niet te wachten op de komst van (nieuwe) eHealth toepassingen.

Helemaal mee eens – Mee eens – Neutraal – Mee oneens – Helemaal mee oneens

Het gebruik van eHealth kan bijdragen aan de veiligheid van cliënten.

Helemaal mee eens – Mee eens – Neutraal – Mee oneens – Helemaal mee oneens

Ik vind het mijn taak om cliënten te ondersteunen bij het gebruik van eHealth.

Helemaal mee eens – Mee eens – Neutraal – Mee oneens – Helemaal mee oneens

Ik ben bang dat het gebruik van eHealth leidt tot minder persoonlijk contact met cliënten.

Helemaal mee eens – Mee eens – Neutraal – Mee oneens – Helemaal mee oneens

Het gebruik van eHealth kan bijdragen aan de zelfredzaamheid van cliënten.

Helemaal mee eens – Mee eens – Neutraal – Mee oneens – Helemaal mee oneens

***Pagina 9: gelegenheid (deel 1)***

1. **Zijn er problemen waar u tegenaan loopt bij het gebruik van eHealth?**

*Het kan hierbij gaan om allerlei soorten eHealth toepassingen (bijv. beeldbellen met cliënten, een elektronisch cliëntendossier, een elektronische medicijndispenser, etc.).*

- Ja, namelijk: …
- Nee
- Niet van toepassing (d.w.z. ik gebruik geen eHealth)

***Pagina 10: gelegenheid (deel 2)***

1. **Stimuleert uw organisatie u om eHealth te gebruiken?**

- Altijd
- Vaak
- Soms
- Zelden
- Nooit

1. **Staat uw organisatie open voor initiatieven van medewerkers omtrent het gebruik van eHealth?**

- Altijd
- Vaak
- Soms
- Zelden
- Nooit

1. **Betrekt uw organisatie medewerkers bij het invoeren van (nieuwe) eHealth toepassingen?**

- Altijd
- Vaak
- Soms
- Zelden
- Nooit

1. **Betrekt uw organisatie cliënten bij het invoeren van (nieuwe) eHealth toepassingen?**

- Altijd
- Vaak
- Soms
- Zelden
- Nooit

***Pagina 11: gelegenheid (deel 3)***

1. **Wat zijn volgens u de belangrijkste factoren die het gebruik van eHealth binnen uw organisatie in de weg (kunnen) staan? Kruis maximaal 3 factoren aan.**

*Het kan hierbij gaan om allerlei soorten eHealth toepassingen (bijv. beeldbellen met cliënten, een elektronisch cliëntendossier, een elektronische medicijndispenser, etc.).*

- Een eHealth toepassing is niet gebruiksvriendelijk.
- Een eHealth toepassing ziet er niet aantrekkelijk uit.
- De gebruiksinstructies van een eHealth toepassing zijn onduidelijk.
- Er wordt geen training aangeboden aan zorgprofessionals voor het gebruik van een eHealth toepassing.
- Er wordt geen training aangeboden aan cliënten voor het gebruik van een eHealth toepassing.
- Er treden technische problemen op bij het gebruik van een eHealth toepassing (bijv. kapotte functies of een trage/instabiele internetverbinding).
- Het gebruik van een eHealth toepassing past niet binnen bestaande werkprocessen van zorgprofessionals.
- Het gebruik van een eHealth toepassing sluit niet aan bij de behoeften van cliënten.
- De privacy van cliënten is onvoldoende gewaarborgd bij het gebruik van een eHealth toepassing.
- De kosten voor het gebruik van een eHealth toepassing zijn te hoog.
- Anders, namelijk: …

1. **Wat kan volgens u helpen om het gebruik van eHealth binnen uw organisatie te bevorderen?**

………………………………………………………………………………………………………………………………………………………………………………………………………………………………………………………………………………………………………………………

***Pagina 8: afsluiting***

**Dit is het einde van de vragenlijst. Hartelijk dank voor uw bijdrage!**
